# Notes and News

**Published:** 1902-04-12

**Authors:** 


### NOTES AND NEWS.

The surprise visit of the King to the Consumption Hospital at Ventnor gave great pleasure. His Majesty made a most careful inspection, admitting that he had in his mind the Sanatorium to be built under his own auspices.

The Prince and Princess of Wales set an admirable example to parents by the way in which they early interest and associate their children with charitable objects. All of their children contribute to King Edward's Hospital Fund, and quite recently they have subscribed to the Kate Greenaway Memorial Cot at the Hospital for Sick Children.

The Hospital Saturday Fund will hold its Annual Collection on October 11th.

Several cases of small-pox have occurred amongst the convicts at Wandsworth prison.

A congress of French alienists and neurologists will be held at Grenoble from August 1st to 8th. Dr. Bonnet, the Secretary-General of the Congress, is Physician of the Asylum of Saint Robert, Isère.

The Medical Officer of Health for the City of London shows that vigilance in respect to the sale of unwholesome food, is very necessary. During four weeks no less than upwards of 8,000 tins of various preserved food had been destroyed as unsound, 56 tons of meat, and 147 samples of food were also condemned.

The late Miss A. B. Miles of Highgate has bequeathed £100 each to the North-West London Hospital, the London Fever Hospital, the Great Ormond Street Children's Hospital, the Mount Vernon Hospital for Consumption, and the National Hospital for Consumption, and £3,000 towards a hospital ship for the Deep Sea Fishermen's Mission, to be called the *Joseph and Sarah Miles* mission ship.

It is reported that in one part of the Punjab the squirrels caught the plague and have become nearly exterminated.

A course of post-graduate clinical demonstrations will be given at the Westminster Hospital during the coming session.

An effort is being made in New York to place all New York State Hospitals for the Insane under the State Commission in Lunacy.

The late Mr. A. Crooke, of St. Margaret's-on-Thames has bequeathed £20,000 to hospitals at his trustee's discretion

Since the outbreak of cholera in Arabia, amongst the Moslem pilgrims, upward of 1,000 deaths have occurred. Strict quarantine will be resorted to on the return of the pilgrims to the outgoing ports.

Anxious relatives of members of Parliament must learn with satisfaction that although the atmosphere of the House of Commons is far from pleasant, it contains no hurtful microbes, so far, that is, as the analyses recently made can decide the matter.

The reports ot the success which has attended the methods adopted in Italy to combat malarial fever are very encouraging. The mechanical method of protecting the dwellings has had excellent results, whilst the beneficial effects of quinine have been so marked as to have induced the Government and many employers to supply it free to their employees.

Dr. Ainley Walker will give a course of twelve lectures on experimental pathology in the Physiological Theatre of Guy's Hospital, commencing May 8th at 4 p.m. and continuing every Thursday at the same hour. The subjects dealt with will be Inflammation, Infection, and Fever. The lectures will be open to all members of the University of London.

One of the reproaches attaching to the methods of local authorities who destroy crowded and insanitary dwellings is, that they do not really improve matters, as they provide no dwellings for the poor they depose, but cause them to find shelter in other already crowded quarters. The Liverpool City Council have done better than this, they have succeeded in supplying a type of dwelling suitable to the means of the very poor who are turned out of condemned buildings. This difficulty has not hitherto been faced elsewhere

At the meeting of the Metropolitan Asylums Board last Saturday, Mr. Scovell, in presenting the report of the hospitals committee, mentioned the case of a male patient who had made his way out of one of the wards of the hospital ship *Castalia* and disappeared. It happened in the middle of the night, and there could be no doubt that the patient got into the water, but it was not certain whether he was drowned or had reached the shore—which was a very short distance—and thus made his way into the country. The matter had been very carefully investigated, and it was impossible to assign blame to anyone.

